# Unusual Acute Pediatric Pyelonephritis Presenting With Cluster Convulsions by Possible Central Nervous System Lesion: A Case Report

**DOI:** 10.7759/cureus.27654

**Published:** 2022-08-03

**Authors:** Masazumi Miyahara, Kyoko Osaki, Katsuya Aoki

**Affiliations:** 1 Department of Pediatrics, Okanami General Hospital, Iga, JPN; 2 Department of Paediatrics, Okanami General Hospital, Iga, JPN; 3 Department of Urology, Nara Medical University, Nara, JPN

**Keywords:** atypical, encephalitis, cluster convulsions, mers, acute pyelonephritis

## Abstract

Acute pyelonephritis is the leading cause of bacterial infection among children. It can be difficult to diagnose early in the disease course owing to non-specific symptoms and physical findings. Recently, some cases of pediatric acute pyelonephritis with mild encephalitis/encephalopathy with a reversible splenial lesion (MERS) have been reported. We describe a case of a six-year-old boy who presented with a high fever and four episodes of cluster convulsions. Despite the absence of leukocyturia and hypo-inflammatory response in the blood, he was diagnosed with acute pyelonephritis by contrast-enhanced computed tomography seven days after onset. The convulsions were not simple febrile convulsions and suggested central nervous system (CNS) lesions, as the patient was older than the usual cut-off age of five years for febrile seizures. This case highlights an unusual presentation and clinical course of a case of pediatric acute pyelonephritis characterized by cluster convulsions and a poor inflammatory response. Furthermore, we strongly consider that the cause of the cluster convulsions may be related to MERS spectrum disorder and emphasize that pyelonephritis can be accompanied by CNS disturbances.

## Introduction

Urinary tract infections (UTIs) are among the most common bacterial infections encountered in childhood. UTIs can be divided into three categories: acute pyelonephritis, acute cystitis, and asymptomatic bacteriuria. Patients with acute pyelonephritis generally present with a high temperature. Acute pyelonephritis should be considered in every child with a fever of unknown etiology; however, diagnosing acute pyelonephritis remains challenging. Herein, we present a case of a child with atypical acute pyelonephritis accompanied by cluster convulsions and a poor inflammatory response. Pyelonephritis, an infection in the abdomen, rarely develops with the central nervous system (CNS) lesion-related symptoms, such as cluster convulsions, as observed in the present case. The only CNS lesion associated with pyelonephritis is mild encephalitis/encephalopathy with a reversible splenial lesion (MERS). Moreover, among MERS-related bacterial infections, pyelonephritis has been reported prominently. Our case is strongly considered to have MERS spectrum disease, distinguishing it from the previously reported MERS-related pyelonephritis in that the disturbance of consciousness did not persist, and neurological disturbance disappeared within 12 h. In this report, we emphasize the importance of recognizing pyelonephritis related to CNS lesions, resulting in an early diagnosis of atypical pyelonephritis.

## Case presentation

A healthy six-year-old boy developed a headache, followed by a high-grade fever at home. The following morning, he experienced an episode of generalized tonic-clonic convulsion (GTC). Although he regained consciousness rapidly, he experienced a second episode of GTC after 3 h in the emergency department and was consequently referred to our hospital. The patient's past medical history was unremarkable, with no convulsion episode before admission, and he was uncircumcised. The family history was also unremarkable, including neurological diseases. 

Physical examination, evaluation of blood, urine and cerebrospinal fluid tests, and provisional diagnosis on admission

The patient was alert on admission, with a temperature, heart rate, and blood pressure of 39.5°C, 135 beats/min, and 110/70 mmHg, respectively. A physical examination did not reveal any remarkable findings. Laboratory investigations revealed a white blood cell count of 25,000 cells/µL (86% neutrophils) and C-reactive protein (CRP) level of 0.37 mg/dL. Leukocyturia was absent, and cerebrospinal fluid examination revealed normal findings (Table 1). We suspected viral infection and administered intravenous fluid therapy. 

The clinical course after hospitalization, diagnosis of the fever origin, and treatment course

**Figure 1:**
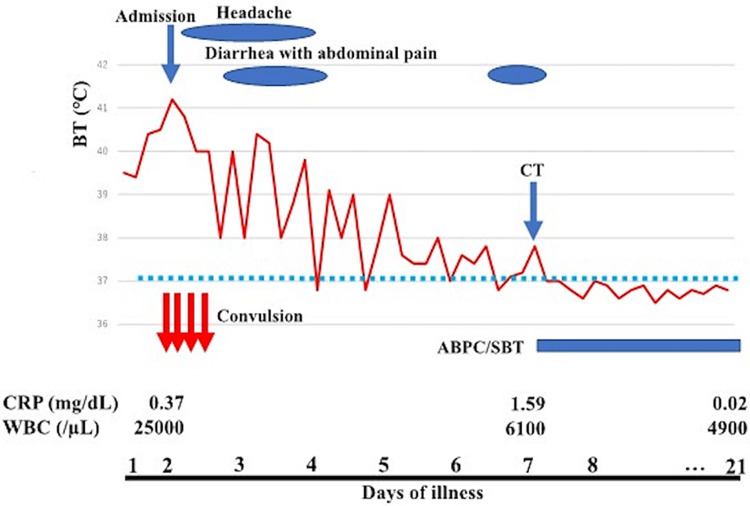
Patient’s clinical course BT, body temperature; CRP, C-reactive protein; WBC, white blood cell count; ABPC/SBT, ampicillin/sulbactam

**Figure 2:**
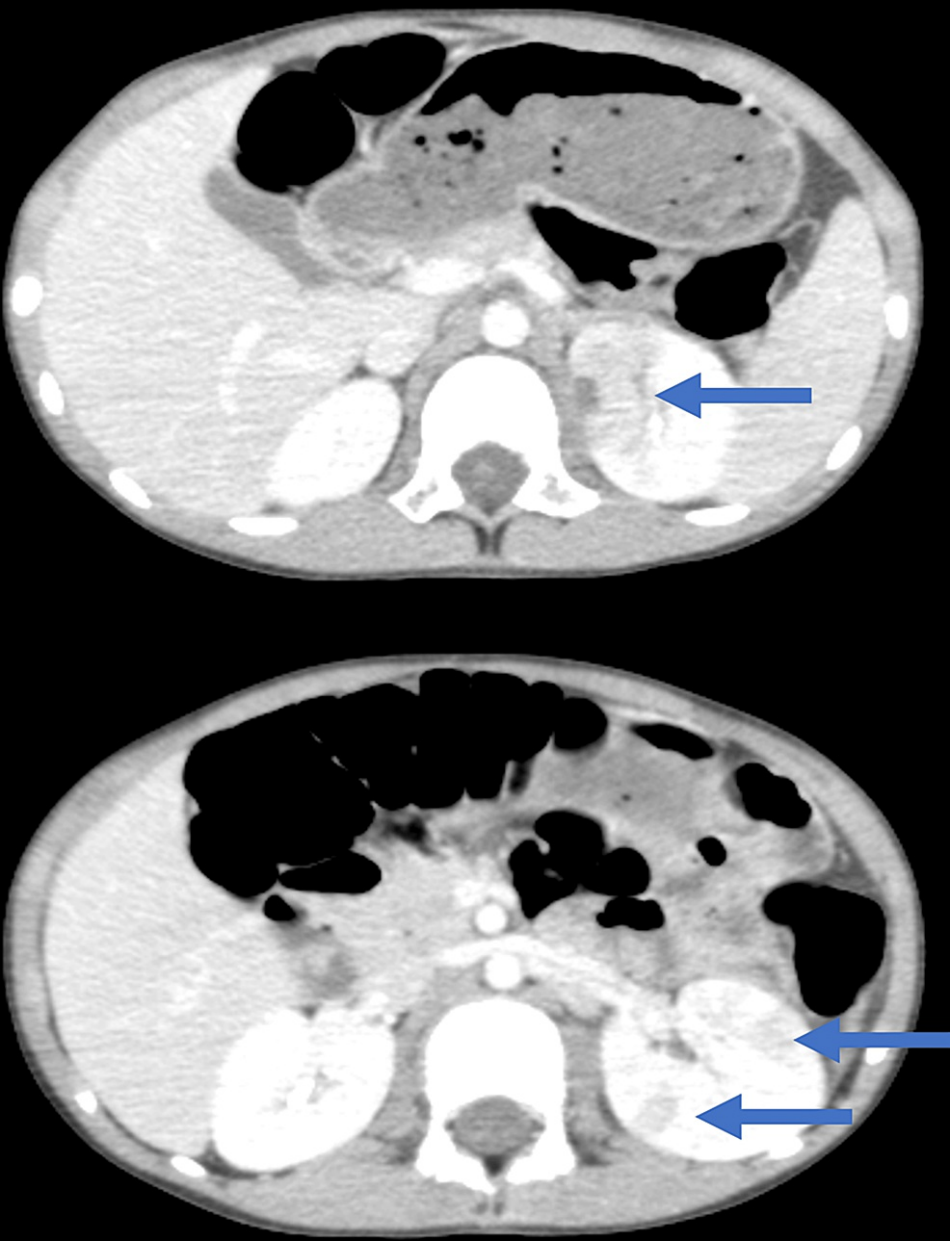
Contrast-enhanced abdominal computed tomography scans showing multiple low-density areas in the left kidney (arrow)

The third and fourth episodes of GTC occurred at 3 and 7 h after the second episode of GTC, respectively; no convulsions occurred after that. A diazepam suppository (6 mg) was administered immediately after the third seizure. During the fourth seizure, intravenous midazolam was required to suppress the seizure; however, all other seizure episodes stopped spontaneously within a few minutes. During the clinical course, there were no meningeal signs. The patient's consciousness level between the convulsions was normal. After the fourth convulsion, there was no recurrence of convulsion, and the state of consciousness remained normal. On the third and fourth days after symptom onset, high-grade fever persisted with maximum temperatures of 40.2℃ and 39.1℃, respectively. The patient occasionally complained of headaches and experienced simultaneous abdominal pain and diarrhea. The symptoms subsided gradually, and the patient resumed normal activities. On the seventh day after symptom onset, the white blood cell count was 6,100 cells/µL (58% neutrophils), and the CRP level was 1.59 mg/dL. Abdominal pain and diarrhea decreased; however, a low-grade fever (37.9℃) persisted (Figure 1). Contrast-enhanced abdominal computed tomography (CT) findings revealed multiple low-density areas in the left kidney, which showed partly wedged-shaped, round, and non-liquefactive lesions, clearly differentiated from the renal cyst or tumor (Figure 2); therefore, he was diagnosed with pyelonephritis. After administering sulbactam/ampicillin, the fever and abdominal symptoms subsided promptly. Urine culture revealed the presence of *Enterococcus faecalis* (10^6^ colony-forming units/mL), which was suspected to be the causative agent. We treated the patient with antibiotics for two weeks, followed by low-dose prophylactic antibiotics; he was discharged on the 21st day of post-symptom onset.

Evaluation of the kidneys and the subsequent course and outcome after discharge

Voiding cystourethrography, performed at three months after his discharge, revealed left vesicoureteral reflux (grade II). After eight months, 99mTc-dimercaptosuccinic acid renal scintigraphy revealed no defect likely to cause renal scarring in both kidneys. The patient showed no relapse of pyelonephritis.

**Table 1:** Laboratory tests data on admission WBC, white blood cell count; RBC, red blood cell count; Ch-E, cholinesterase; ALP, alkaline phosphatase; AST, aspartate aminotransferase; ALT, alanine aminotransferase; LDH, lactate dehydrogenase; CPK, creatine phosphokinase; T-Cho, total cholesterol; BUN, blood urea nitrogen; Crea, creatinine; CRP, C-reactive protein

Laboratory parameters	Patient value	Reference range
Peripheral blood test		
WBC	25000 /μL	4000–9000
neutrophil	86%	39–81
lymphocyte	10%	16–50
monocyte	4%	2–10
RBC	483 ×10^4^/μL	400–520 ×10^4^
Hemoglobin	13.3 g/dL	13.0–17.0
Hematocrit	37.7%	38.0–49.0
Platelet	30.3 ×10^4^/μL	12.0–44.0 ×10^4^
Serum biochemical test		
Total protein	7.1 g/dL	6.5−8.5
Albumin	4.7 g/dL	4.1−5.3
Total bilirubin	0.6 mg/dL	0.2−1.2
Ch-E	414 U/L	214–466
ALP	282 U/L	38–113
AST	32 IU/L	10–35
ALT	17 IU/L	10–35
LDH	329 U/L	110–225
CPK	109 IU/L	50–200
T-Cho	181 mg/dL	150–219
BUN	10.7 mg/dL	9.0–22.0
Crea	0.30 mg/dL	0.50–1.10
Sodium	134 mEq/L	138–145
Potassium	4.0 mEq/L	3.4–4.7
Chloride	99 mEq/L	99–108
CRP	0.37 mg/dL	0.00–0.30
Urinalysis		
Specific gravity	1.016	1.006–1.020
pH	7.0	4.5–7.5
Protein	negative	
Glucose	negative	
Ketone	negative	
Sediment		
WBC	1–4/HPF	1–5
Cerebrospinal fluid test		
Protein	23 mg/dL	10–40
Glucose	99 mg/dL	50–75
Cell	1/μL	0–5
Culture		
blood	no growth	
cerebrospinal fluid	no growth	
urine	*Enterococcus faecalis* 10^6 ^CFU/ml	
stool	Escherichia coli	
	Enterobacter cloacae	
	Enterococcus species	
	Bacillus subtilis	
Rapid antigen test by immunochromatography on stool		
Vero toxin	Negative	
Rotavirus	Negative	
Adenovirus	Negative	

## Discussion

UTIs affect approximately 7%-8% of children during the first eight years of life [1]. Pyelonephritis requires prompt diagnosis and treatment; delayed or inadequate treatment could result in renal scarring, leading to hypertension and reduced kidney function. Pediatric pyelonephritis usually manifests as high-grade fever or gastrointestinal symptoms, including abdominal pain, vomiting, and diarrhea. However, the symptoms are non-specific, and early diagnosis depends on elevated inflammatory markers on blood tests and leukocyturia. However, at least 10% of children with UTIs do not present with leukocyturia, leading to a misdiagnosis, especially if caused by bacteria other than *Escherichia coli* [2]. In the absence of leukocyturia, imaging tests are necessary for diagnosis. An ultrasound of the urinary tract neither proves nor excludes the presence of pyelonephritis. An abdominal contrast-enhanced CT is the most reliable method to detect pyelonephritis; however, it may result in unnecessary radiation exposure in children. Accordingly, unless pyelonephritis is strongly suspected, a CT should not be performed [3,4]. We aimed to determine the presence or absence of pyelonephritis as the fever origin as soon as possible. For accurate diagnosis of pyelonephritis, we thought that abdominal echo findings may often be false negative; thus, we gave priority to CT examination. In our case, the patient experienced cluster convulsions with high-grade fever, an infrequent manifestation of pyelonephritis. The convulsions were similar to febrile convulsions. However, as the first seizure in his life occurred at a relatively late age (six years), the convulsions appeared in clusters, and the diazepam suppository, which is extremely effective in preventing febrile convulsions, could not prevent the fourth seizure; the patient's convulsions were not considered as typical febrile convulsions, but strongly suggested the presence of CNS lesions. Neurological abnormalities, such as MERS associated with pyelonephritis, have been previously reported as possible causes of cluster convulsions [5]. MERS is a clinical and radiological syndrome first proposed by Tada et al. in 2004 [6]. The diagnosis of MERS was based on the criteria proposed by Hoshino et al., which required the following: (1) onset of neuropsychiatric symptoms, such as abnormal speech or behavior, impaired consciousness, and seizure within one week after the appearance of fever; (2) complete recovery without sequelae, typically within 10 days after the onset of neuropsychiatric symptoms; (3) high signal intensity lesion on diffuse-weighted imaging in the splenium of the corpus callosum in the acute stage, with mild T1 and T2 signal changes; (4) lesion may involve the entire corpus callosum and symmetrical cerebral white matter; and (5) neuropsychiatric symptoms that continue for >12 h. However, even if the neurological symptoms disappear within 12 h, it is considered the same spectrum of MERS [7]. In the current case, our patient's consciousness recovered swiftly between convulsions, and he presented no apparent neurological abnormalities. No abnormalities were observed in the cerebrospinal fluid; therefore, there was no obvious encephalitis or meningitis. The convulsions disappeared within 12 h after the onset of symptoms. No abnormalities causing convulsions were found besides pyelonephritis. Unfortunately, the patient did not undergo a head magnetic resonance imaging or CT scan. As his consciousness level was normal between the convulsions and after the fourth episode of convulsion, normal CSF findings and hypo-inflammatory reaction on the blood, including the convulsions, seemed to be attributed to febrile seizures initially. Hence, we could not comprehend that the cause of convulsions was in the head. Moreover, there was little awareness of MERS. After the observation of pyelonephritis, while considering the cause of the cluster convulsions, we learned that there were some reports of pyelonephritis with MERS and thought that this might have been the cause. At that time, we considered that it was too late to perform imaging tests for the patient using sedatives, which were needed for the patient. However, MERS spectrum disorder is the only possible explanation for this patient's convulsions. The patient's seizures cannot be explained as febrile seizures and should not be considered as febrile seizures. A total of 983 cases reported acute encephalopathy in Japan between 2007 and 2010. Among the 153 cases of MERS, influenza virus (34.4%), rotavirus (11.7%), mumps virus (3.9%), herpesvirus-6 (2.0%), and bacterial (3.3%) infections [7] were observed. Among bacterial infectious diseases, acute pyelonephritis is the most frequent cause of MERS in children. Further, only MERS has been reported as a CNS lesion associated with pyelonephritis. Our case is strongly considered to have MERS spectrum disease, distinguishing it from the previously reported MERS-related pyelonephritis in that the disturbance of consciousness did not persist, and neurological disturbance disappeared within 12 h. A bacterial infection in the kidney might have affected the CNS through an unrecognized mechanism. In addition, it was challenging to diagnose pyelonephritis as the cause of fever, as leukocyturia was absent, and a poor inflammatory response was observed in blood tests. There is no robust evidence regarding inflammatory markers for diagnosing pyelonephritis. However, repeated low levels of inflammatory markers, such as CRP (<2 mg/dL) or procalcitonin (<0.5 μg/L) make the diagnosis of pyelonephritis less likely [8]. We did not measure the procalcitonin level in our case, but the CRP level was repeatedly low. This evidence made the initial diagnosis of pyelonephritis unlikely. Although the reason for the poor inflammatory response is unknown, *E. faecalis* might have a poor pro-inflammatory potential because of its modulation of the host immune response [9]. This could be the cause of the hypo-inflammatory response on blood examination.

This study reports an unusual presentation of cluster convulsions with a common infection, acute pyelonephritis, and we have highlighted a possible rare association with MERS spectrum disorder, which could occur in diseases with only convulsions without other neurological disturbances. The existing literature enlists MERS as a rare manifestation of pediatric pyelonephritis. The major limitation of this report was the lack of brain imaging to support the underlying claimed association with MERS. Therefore, the association between our case and MERS spectrum disorder can only be presumptive. Nonetheless, this is an important differential diagnosis in this case because the cases of acute pyelonephritis with the presentation of neurological symptoms, including MERS, have been reported, and could be a challenge or a pitfall in the early diagnosis in children.

## Conclusions

This is a case report of an extremely atypical presentation of cluster convulsions and clinical course with a common infection, acute pyelonephritis, in which we have tried to highlight a possible rare association with MERS spectrum disorder. The latter could occur in diseases with only convulsions without other neurological disturbances. It is noteworthy that bacterial infections in the kidney could be associated with the CNS.

The findings of this case provide vital information on acute pyelonephritis and emphasize the importance of recognizing atypical manifestations, cluster convulsions, and poor inflammatory response in pediatric patients with pyelonephritis for clinicians worldwide. This knowledge will assist in the early diagnosis and timely treatment of acute pediatric pyelonephritis.
